# ASVmaker: A New Tool to Improve Taxonomic Identifications for Amplicon Sequencing Data

**DOI:** 10.3390/plants12213678

**Published:** 2023-10-25

**Authors:** Clément Plessis, Thomas Jeanne, Antoine Dionne, Julien Vivancos, Arnaud Droit, Richard Hogue

**Affiliations:** 1 Institut de Recherche et de Développement en Agroenvironnement, Québec, QC G1P 3W8, Canada; 2 Computational Biology Laboratory, CHU de Québec—Université Laval Research Center, Québec City, QC G1V 4G2, Canada; 3 Laboratoire d’Expertise et de Diagnostic en Phytoprotection, Ministère de l’Agriculture, des Pêcheries et de l’Alimentation du Québec (MAPAQ), Québec City, QC G1P 3W6, Canada

**Keywords:** taxonomic assignment, QIIME2, ASV-specific database, pre-trained classifiers, public reference databases

## Abstract

The taxonomic assignment of sequences obtained by high throughput amplicon sequencing poses a limitation for various applications in the biomedical, environmental, and agricultural fields. Identifications are constrained by the length of the obtained sequences and the computational processes employed to efficiently assign taxonomy. Arriving at a consensus is often preferable to uncertain identification for ecological purposes. To address this issue, a new tool called “ASVmaker” has been developed to facilitate the creation of custom databases, thereby enhancing the precision of specific identifications. ASVmaker is specifically designed to generate reference databases for allocating amplicon sequencing data. It uses publicly available reference data and generates specific sequences derived from the primers used to create amplicon sequencing libraries. This versatile tool can complete taxonomic assignments performed with pre-trained classifiers from the SILVA and UNITE databases. Moreover, it enables the generation of comprehensive reference databases for specific genes in cases where no directly applicable database exists for taxonomic classification tools.

## 1. Introduction

### 1.1. Amplicon Sequencing

High-throughput sequencing approaches and, more specifically, amplicon sequencing allow the generation of a large diversity of genetic variants. They represent the relative composition of a microbial group in an environmental DNA (eDNA) sample. This molecular approach is dependent on the specific primers used [1,2], and several systems allow us to analyze the diversity of bacteria [3], fungi [4,5], and other microbial groups [6,7] detected in eDNA samples.

Computer processing of high-throughput sequencing data is essential to obtain reliable and high-quality results. To reduce the influence of sequencing errors, the first strategy has been to generate similarity clusters by defining operational taxonomic units (OTU) at a similarity threshold of 97%. This approach was suitable for early sequencing technologies (e.g., 454). Over the past decade, significant advancements have been made in the tools and methods used to process this type of data. These advancements aim to reduce sequencing errors’ impact and enhance downstream analyses’ accuracy. New tools such as DADA2 [8] allow the generation of genetic variants with high accuracy [9]. The algorithms use machine learning approaches to optimize sequencing error handling. With this type of processing, amplicon sequence variants (ASV) can be obtained.

### 1.2. Public Reference Database and Taxonomic Limitations 

Taxonomic classification is an important step in sequence processing. Representative ASVs can be compared with reference sequences. Historically, there are important reference databases such as Genbank [10], DDBJ [11], and the European Nucleotide Archive (ENA) that accumulate all deposited sequencing data. These reference data are not necessarily well-balanced between species, and there are many problematic annotations. Several research groups have developed curated and aligned reference databases to facilitate the processing of high-throughput sequencing (HTS) data to facilitate taxonomic assignment. The SILVA [12], Greengenes [13], RDP [14], WarcupRDS [15], and UNITE [16] databases are widely used in microbial ecology for consensus sequence identification, but several problems remain. Each taxonomic assignment is linked to an accuracy that depends on the amount of data available in the reference databases. Additionally, a significant ratio of microbial diversity remains unknown due to the inability to cultivate several microorganisms [17]. As a result, some taxonomic assignments remain imprecise. Moreover, in cases where species exhibit high genetic similarity within the amplified region, classifiers can only reach a consensus at the genus level. Consequently, the application of HTS approaches for species identification related to some microbial genera is limited.

### 1.3. Available Tools to Assign Taxonomy

Bioinformatics tools for taxonomic identification are becoming more and more powerful. Some classifiers use machine learning approaches like “SKlearn” to improve classification and speed up data processing [18], while other more conventional approaches allow more parameter settings, for example, Vsearch [19] or DADA2 [8]. To make the HTS data usable and to facilitate result presentation, a consensus assignment is provided for each previously identified ASV. An accuracy calculation is possible using a pre-trained classifier, but the taxonomic assignment decision is conservative. This procedure is generally suitable for most applications in microbial ecology. However, there are limitations when it comes to identifying non-cultivated species and genera having exact similarities within the targeted gene. Finally, more specific tools propose treatments to improve the accuracy of taxonomic assignment [20]. These tools are also very dependent on the available reference databases generated by taxonomist research groups. There is a lack of tools to easily generate and use more specific reference databases for less studied genes (e.g., EF1-alpha, Beta tubulin, cytochrome oxidase II).

Here, a new tool that allows the creation of specific and usable ASV-specific reference databases for HTS data purposes is presented. This provides information on all possible identifications for each ASV and contributes to a better taxonomic assignment.

## 2. Materials and Methods

### 2.1. Environment

ASVmaker is an open-source tool available at. This is a Python-based tool that is completely interoperable. It can be deployed using the Python Package Index (PyPi) Python-based tool. We recommend using it by command line. The installation and use procedure is described in the tool’s GitHub repository, available at the following address: https://github.com/cplessis/ASVmaker (accessed on 1 August 2023).

### 2.2. ASVmaker Functionalities

#### 2.2.1. Structure

**Figure 1:**
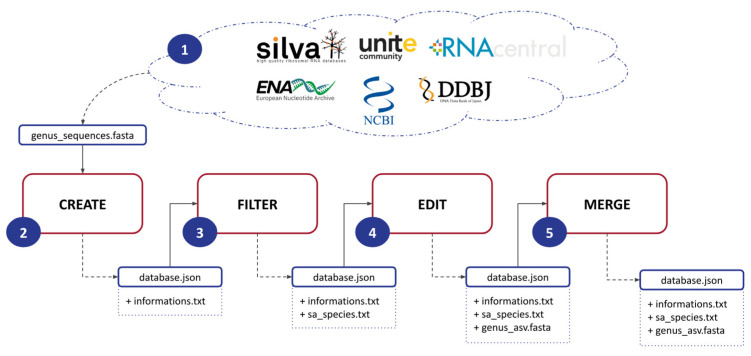
Schematic of the five steps of ASVmaker’s process. (1) Download the FASTA file for one genus from a general database, (2) produce the database for a specific genus and primers, (3) filter redundant amplicons or unwanted taxonomy, (4) produce shared amplicon (SA) groups, and (5) prepare facultative merging of specific genus ASV databases from different general databases.

ASVmaker is designed to be used by modules (Figure 1). (1) The first step involves downloading a FASTA file for a specific genus of interest from a general database: Silva, Unite, RNAcentral, ENA, NCBI, or DDBJ. This file contains the genomic data necessary for subsequent analysis. (2) Next, ASVmaker enables the creation of a genus-specific database using the downloaded FASTA file. Each sequence lineage is verified by accession number through the European Nucleotide Archive API if possible and through the NCBI Entrez API if the ENA one does not match. Users must specify primers to be used during the simulation of the amplification process, allowing for precise targeting of the desired genomic regions and ASV creation. (3) To enhance the quality and specificity of the analysis, ASVmaker provides the functionality to filter out redundant amplicons and exclude unwanted taxonomy. Redundant amplicons are ASVs sharing the same taxonomy. Unwanted taxonomy or species that are not of interest (e.g., “sp.” or “aff.”) can also be filtered out, ensuring a more focused analysis of the target genus. (4) ASVmaker creates shared amplicon (SA) groups, which involve clustering identical ASVs with different taxonomies. This grouping allows for a comprehensive understanding of the taxonomic diversity within the selected ASV, providing valuable precisions into the composition and dynamics of microbial communities. (5) Moreover, ASVmaker offers the option to merge ASV-specific databases from different general databases, providing flexibility to combine data from various sources. When two reference databases for the same genus are built from two different FASTA files, it is possible to merge them. This step creates new SA groups if necessary and eliminates duplicates. This merging process allows for a more comprehensive dataset, enabling comparative analysis and broader insights into the studied genus.

#### 2.2.2. Taxonomy

A taxon is defined as the most precise taxonomic description that can be obtained for a sequence variant. With the currently available tools, a variant with a different possible taxonomy is, by default, assigned to the consensus taxonomy at a truncated level (e.g., “GenusName_spp.”). This results in the loss of crucial information. A solution to this problem is to assign a group of species sharing the same amplified sequence as a taxon. In this case, the amplicon is defined as “Shared Amplicon” (SA). The taxon of an ASV related to a single species will, therefore, be “GenusName_SpeciesName.” The taxon of an ASV related to several species will be “GenusName_SAn,” where “n” is the sequential number of the SA in the database. The choice to group these sequences under the name of the SA is an important step to avoid losing information on genetic variability. Thus, the identification by HTS will return a maximum of answers to the user without passing by a consensus attribution. Hence, it is possible to attribute a taxonomic identification by grouping very similar sequences. The SA groups give the same information as Blast at 100% identity on same-length sequences for multiple species but stored in the sequence taxonomy. ASVmaker does not rely on any specific algorithm, unlike other classifiers.

#### 2.2.3. Amplicon

To create an ASV-specific database, a simulation of amplification must be performed on all the sequences to select the amplifiable fragments. The original amplification system was based on the PCR function of the Python package Pydna [21]. However, a custom module was created because this package does not offer customizable parameters for primer mismatch tolerance. This module uses local primer alignment scores on a given sequence. To favor the positions where the primer can attach, the calculation of the scores favors match and mismatches rather than gaps: match +1, mismatch 0, open-gap −1, extend-gap −0.5. The “sense” leader is directly aligned to the sequence, and the position with the highest score is saved. Then, the complementary strand of the reverse primer is synthesized before it is also aligned to the sequence. If the alignment score of the two primers passes the threshold set by the user, then the amplicon is generated on the primer positions with or without end primers (as desired). An amplicon is created only if the last three bases at the 3’ ends do not contain any mismatches.

#### 2.2.4. Usage

When seeking to identify an ASV from the amplification of a large microbial group (e.g., bacteria or fungi), an ASV-specific database generated by ASVmaker can be used on ASVs that have generated an initial identification at the genus level. This constitutes a case of double identification, firstly by a general database such as Silva or UNITE and secondly with the specific one from ASVmaker. For other applications, when dealing with ASVs generated by the amplification of a specific genus (e.g., *Fusarium* for the EF1-alpha gene), the specific database generated by ASVMaker can be used directly. In all cases, the taxonomic assignment with the specific reference database must be used with 100% alignment and 100% coverage.

### 2.3. Creation of a New Database

To evaluate the performance of ASVmaker, ASV-specific reference databases have been generated. It was chosen as a microbial genus that may include plant pathogens. This application in plant pathology is not the only one, but it was chosen because we are involved in a project to evaluate the potential of HTS for the identification of several plant pathogenic organisms. For bacteria, the targeted genera have been *Erwinia, Streptomyces, Pseudomonas,* and *Xanthomonas*. The fungal genera have been *Colletotrichum, Septoria, Ustilagi,* and *Verticillium*. These ASV-specific databases can be combined with the taxonomic assignment with the pre-trained classifiers (SILVA version 138 or UNITE version 8.3) to improve the species-level identification.

An additional ASV-specific database has been generated to present an example of a direct and specific amplification targeting a non-ribosomal gene. The *Fusarium* elongation factor alpha gene was targeted. In the 3 targeted examples, we used ASVmaker with the primers described in Table 1 and sequences from queries from UNITE for fungal genera, SILVA for bacterial ones, and RNAcentral for both. Since there is no sequence of EF1α in the UNITE database, the sequences available from the ENA database were downloaded.

### 2.4. Application on Environmental Samples

To provide examples of applications, plant samples from a large study focused on the potential for identifying plant pathogenic organisms using HTS were used. These examples compared the identification process using public reference databases (SILVA, UNITE) to the dual identification method based on the reference database generated with ASVMaker.

#### 2.4.1. Sample and DNA Extraction

Plant tissues were collected by the Ministère de l’Agriculture, des Pêcheries et de l’Alimentation du Québec (MAPAQ) plant pathologists based on specific disease symptoms. The fresh tissues were homogenized, and 0.2 g were used for DNA extraction. DNA extractions were performed with the DNeasy Plant Mini Kit (Qiagen, Mississauga, ON, Canada) according to the manufacturer’s instructions. Each DNA pellet was suspended in 100 μL of sterile molecular-grade deionized water. The quality and quantity of the DNA extracts were evaluated by spectrophotometry using a Biophotometer (Eppendorf, Mississauga, ON, Canada) with readings at 260, 280, 230, and 320 nm. 

#### 2.4.2. Amplicon Sequencing

Prokaryote and fungal diversity were assessed by HTS as described [26], using 515FB and 926R primers and BITS-ITS1 and B58S3 primers, respectively, for bacteria and fungi. Specific *Fusarium* spp. amplification was performed using the primers Fa-150 and Ra-2, targeting the elongation factor 1-alpha gene (Table 1). Briefly, a two-step dual-indexed PCR approach was specifically designed for Illumina instruments by the Plateforme d’analyses génomiques (IBIS, Université Laval, Quebec City, QC, Canada) was performed. Indexed PCR products were purified, checked for quality on a DNA7500 Bioanalyzer chip (Agilent, Santa Clara, CA, USA), and then quantified spectrophotometrically using the Biophotometer with a G1.0 μCuvette. Barcoded amplicons were pooled in equimolar concentrations for sequencing on the Illumina MiSeq platform using a 2 × 300 bp sequencing kit.

#### 2.4.3. Bioinformatic Analysis

Raw MiSeq sequences (FASTQ) were filtered under the QIIME2 platform [27] using the DADA2 plugin [8] filtration approach for determining amplicon sequence variants (ASV). For fungi sequences of the ITS1 region, primers were previously removed with the Cutadapt tool [28]. 

Taxonomic assignments were carried out using a classification approach with the sklearn function in the q2-feature-classifier plugin [18] and pre-trained classifiers from the SILVA (version 138) and UNITE (version 8.3) databases for bacteria and fungi, respectively. The secondary assignment was generated with 100% similarity identification using the ASV-specific database obtained with ASVmaker. For the specific EF1α gene, the ASV-specific database generated from the EF1α sequences was used directly.

## 3. Results

### 3.1. ASV Specific Database for 16S rRNA, ITS and EF1α Gene

**Figure 2:**
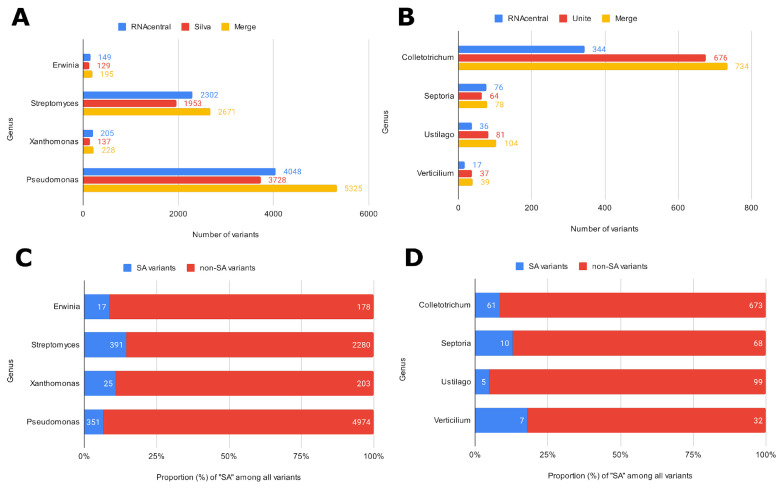
Number of variants retained according to the data source and ASV combination for bacterial genus (**A**) and targeted fungal genus (**B**). Number of unique variants (non-SA) or variants with at least one different taxonomic identification (SA) for bacterial genus (**C**) and fungal genus (**D**).

Three ASV-specific databases were created to showcase ASVmaker use cases (all code available in the “data and code availability” section). Two of these were designed to complete the analysis with the Silva and UNITE pre-trained classifiers. Four bacterial genera and four fungal genera were chosen to present a simple and complex case study for each microbial group targeted in the phytopathological application. The raw sequences were then retrieved from the Silva database for bacterial genus and primers targeting the 16S region, from the Unite database for fungal genus and primers targeting the ITS region, and from the RNAcentral database for both. The tool can concatenate specific ASV bases from different generalist bases (Figure 2). For all the genera studied, ASVmaker made it possible to increase the number of variants by concatenating the two generalist bases. The developed tool enables us to better characterize identical variants with different taxonomies (SA). These variants represent, on average, 10% of the ASVs of the four bacterial genera and 11% of the fungal ASVs.

**Figure 3:**
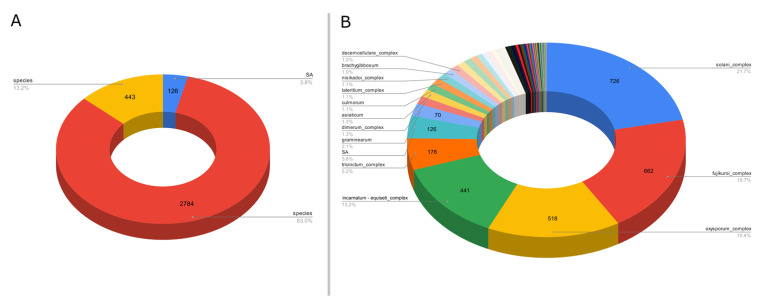
(**A**) Proportion of variants taxonomically assigned to species, species complex, or SA from 3353 variants *Fusarium* EF1α database created from 43,509 sequences retrieved from the ENA. (**B**) Major taxa proportion among 3353 variants in the *Fusarium* EF1α database created from 43,509 sequences retrieved from the ENA.

For the third example, we targeted the gene EF1α to evaluate the *Fusarium* species diversity. This ASV-specific database was created from sequences present in a non-specialized generalist database to reach a better diversity of sequences for less studied genes, the ENA (European Nucleotide Archive). However, these databases may have taxonomic assignment errors on their sequences, unlike databases such as Silva and UNITE, which are more accurate. A total of 43,509 raw sequences were retrieved from the ENA website. After processing with ASVmaker, 3353 unique variants were identified, including 126 SA variants and 2784 species complex variants (Figure 3A). A total of 77 unique species taxa (including species complex) and 126 SA taxa were isolated in the *Fusarium* EF1α ASV specific database for a total of 203 possible taxonomic attributions. Most of the variants of the created specific database targeting the gene EF1α are species complex taxa or SA taxa (Figure 3B).

### 3.2. Environmental Samples Application

One possible application of ASVmaker is to provide an additional level of information aiming at plant pathogen identification. The use of high-throughput sequencing could complement or enhance phytopathologists’ ability to detect plant diseases. As part of a large-scale study in collaboration with the MAPAQ’s phytopathologists, several hundred diseased plants were tested, and plant pathogens identification was obtained using conventional methods (Microscopic, qPCR) and with HTS w compared. To illustrate the benefits of using the databases generated with ASVmaker, samples that could be used in the five following situations (code C1 to C5) were identified:

- C1: Confirmation of the identification obtained with pre-trained classifiers (from the Silva/UNITE databases) with the ASV-specific database;
- C2: Precision increase to the species level with the ASV-specific database;
- C3: Change of species identification with the ASV-specific database;
- C4: Precision obtained with the ASV-specific database with a few species possibilities (simple case);
- C5: Precisions obtained with the ASV-specific database with several species possibilities (complex case).

Table 2 shows the results obtained for the taxonomic identification of the selected cases and according to the overall diversity of bacteria, fungi, and fusarium-specific diversity determined by EF1α gene diversity. A first interpretation illustrates that, whatever the microbial group, it can be easy or more complex to make a good taxonomic identification with HTS data. It is, therefore, not possible to generalize about identification problems. On the other hand, the cases selected for bacteria present more problems compared to fungi. Without being exhaustive, identifications are more problematic for *Pseudomonas, Xanthomonas,* and *Streptomyces*, and the number of possible species can vary widely (from a few species to 44). However, ASV-specific databases can improve taxonomic identifications, such as Cases 3 and 5 for *Streptomyces*, or enable identification at the species level, such as Case 3 for *Erwinia tracheiphila*.

On the other hand, the taxonomic identification improvement provided by ASV-specific databases can be used to discriminate variants potentially associated with a given species. In a case when *Pseudomonas syringae* is targeted, it is possible to discard some variants that do not present this species in the shared amplicon list. 

In the case of fungi, identifications are generally more accurate. Examples in Table 2 illustrate these observations with the identifications of Colletotrichum, Ustilago, and *Verticillium*. For *Colletotrichum*, the secondary identification detailed a more problematic identification with three possible species against one with the pre-trained classifier (Case 7) or change the species identification (Cases 9 and 10). This example highlights the problem of dataset training size of the classifiers. The same observations are reported for *Verticillium* with two possible species identified with ASV-specific database (cases 7, 9, and 10) and for a more problematic case with *Septoria* (Case 9). However, for *Ustilago*, which was a simple case, the same identification was obtained with both databases. The same observations generally apply to other genus.

Samples analyzed for the *Fusarium*-specific gene (EF1α) generally showed a very good level of identification. Unlike the application for bacteria and fungi, the results for the *EF1α* gene allow direct identification. The identifications obtained by HTS and ASV-specific databases can be corroborated with microbial isolations on selective media. In all samples where *Fusarium* spp. was identified by isolation, it was possible to obtain identification by HTS. On the other hand, species identifications may be different or expressed by different names or species complexes. Identifications coupled with relative abundance enable the identification of variants detected in the same sample and to assess their respective representation. Except for the *Fusarium*_SA89 and *Fusarium*_SA93 variants, which have 2 and 6 possible identifications, respectively, all other variants are identified as species or species complex.

## 4. Discussion

ASVmaker is a specialized tool that addresses various application gaps using amplicon sequencing data. It offers additional taxonomic information to confirm species identification or improve identification challenges encountered with conventional classifiers.

While many existing tools aim to enhance taxonomic attributions through database generation, either by refining existing databases or employing more powerful algorithms [29,30,31], ASVmaker is more specifically designed to target a particular genus or a list of genera, adapting accordingly to the primers used in sequencing library preparation. 

ASVmaker can also be used to improve a specific already-generated ASV database. The merge function allows the addition and integration of additional sequences into a newly documented structure. However, it is important to note that ASVmaker is not able to treat multiple genera simultaneously. In this study, it was tested on 10 bacterial genera and 38 fungal genera. As the tool does not address inter-genus issues, employing it as a subsequent step following taxonomic assignment with a pre-trained classifier is crucial.

Additionally, ASVmaker can be used to generate a genus-specific ASV reference database for non-ribosomal genes. The results with the *EF1α* gene showed that ASVmaker can improve taxonomic assignment directly compared to other studies using conventional classifiers [32]. Identifying species with conventional classifiers can be difficult due to conflicts with multiple taxonomies for a single variant. However, ASVmaker can isolate and retain this information in the taxonomic assignment. It is feasible to prepare similar reference databases for other genes of interest in microbial ecology, such as beta-tubulin or cytochrome oxidase II.

Presently, ASVmaker is restricted to data generated from the Illumina platform, as it requires high-quality sequences for successful implementation. Therefore, using an ASV-specific database on sequences from sequencing approaches involving Oxford Nanopore Technology (ONT) is not feasible. Conversely, it may exhibit promising performance for approaches such as Pacbio or other techniques generating high-quality sequences.

## 5. Conclusions

By allowing users to easily prepare their own ASV-specific database and complete the taxonomic annotation from public pre-trained classifiers, ASVmaker will enable researchers in microbial ecology to improve taxonomic identifications for specific microbial genera. The use of ASV-specific databases does not guarantee precise microbial species identification but clarifies potential issues with pre-trained classifiers. ASVmaker also proves to be a powerful tool for constructing a genus-specific ASV reference database for non-ribosomal genes. It was tested on the *EF1α* gene, and it achieved highly interesting performance, obtaining species-specific identifications in most cases. This tool has a wide range of applications, including plant pathology, studying the results of microbial inoculants and biostimulants, as well as applications in biomedical research.

## Figures and Tables

**Table 1:** List of primers used to produce the ASV-specific database and for the amplifications performed on the environmental DNAs.

Microbial Group	Target	Region	Forward Primer	Reverse Primer	Reference
Bacteria	16S	V4V5	515FB: GTGYCAGCMGCCGCGGTAA	926R: CCGYCAATTYMTTTRAGTTT	[22,23]
Fungi	ITS	ITS1	BITS: ACCTGCGGARGGATCA	B58S3 GAGATCCRTTGYTRAAAGTT	[24]
Fusarium	EF1α	EF1α	Fa-150: CCGGTCACTTGATCTACCAG	Ra-2: ATGACGGTGACATAGTAGCG	[25]

**Table 2:** Detailed results of the best-taxonomic identifications obtained with the pre-trained classifiers from the SILVA and UNITE databases and with the ASV-specific database created with ASVmaker for the selected samples. The table shows three sections for the amplification system targeting bacteria, fungi, and specifically *Fusarium* spp. using the *EF1α* gene.

Sample Code	Crop	Diagnostic (Conventional)	Code	Best Taxonomic Identification by Pre-Trained Classifiers (SILVA/UNITE)	Conf.	Complementary Identification with ASV-Specific Database	Shared Amplicon (SA)	Relative Abound. (%)
**Bacterial amplification—** **16S rADN**								
Case1	Squash	*Pseudomonas_syringae*	C5	*Pseudomonas*	1	*Pseudomonas*_SA46	35 species	8.55
			C5	*Pseudomonas*	1	*Pseudomonas*_SA63	28 species	30.89
Case2	Cabbage	*Xanthomonas campestris*	C5	*Xanthomonas*	0.997	*Xanthomonas*_SA1	27 species	57.35
Case3	Squash	*Erwinia_traqueiphila*	C1	*Erwinia tracheiphila*	1	*Erwinia tracheiphila*		31.03
			C5	*Pseudomonas*	1	*Pseudomonas*_SA22	44 species	0.06
			C4	*Streptomyces*	1	*Streptomyces*_SA167	*S.roseirectus* *S.niveiscabiei* *S.acidiscabies*	0.06
Case4	Cabbage	*Xanthomonas campestris*	C5	*Xanthomonas*	0.997	*Xanthomonas*_SA1	27 species	63.11
Case5	Wheat	*Xanthomonas campestris*	C5	*Pseudomonas*	1	*Pseudomonas*_SA22	44 species	0.26
			C5	*Xanthomonas*	0.997	*Xanthomonas*_SA1	27 species	3.97
			C5	*Xanthomonas*	1	*Xanthomonas*_SA3	15 species	49.50
Case5	Potato	*Streptomyces_scabies*	C5	*Pseudomonas*	1	*Pseudomonas*_SA39	30 species	0.18
			C5	*Pseudomonas*	1	*Pseudomonas*_SA46	35 species	0.92
			C5	*Streptomyces*	0.999	*Streptomyces*_SA63	25 species	7.19
			C2	*Streptomyces*	1	*Streptomyces scabrisporus*		0.36
**Fungal amplification—** **ITS1**								
Case7	Potato	*Colletotrichum*, *Dickeya* sp., *Fusarium*, *Pythium*, *Verticillium*	C4	*Colletotrichum coccodes*	0.998	*Colletotrichum*_SA61	*C.nigrum* *C.coccodes* *C.gloeosporioides_complex*	24.46
			C4	*Verticillium nubilum*	0.998	*Verticillium*_SA1	*V.longisporum* *V.dahliae*	17.81
Case8	Corn	*Ustilago_maydis*	C1	*Ustilago maydis*	1	*Ustilago maydis*		13.32
Case9	Melon	*Verticillium_dahliae*	C3	*Colletotrichum fuscum*	1	*Colletotrichum destructivum* complex		0.10
			C5	*Septoria epilobii*	0.93	*Septoria*_SA3	38 species	0.01
			C4	*Verticillium nubilum*	0.998	*Verticillium*_SA1	*V.longisporum* *V.dahliae*	12.51
Case10	Melon	*Verticillium_dahliae*	C3	*Colletotrichum fuscum*	0.998	*Colletotrichum destructivum complex*		0.01
			C4	*Verticillium nubilum*	0.998	*Verticillium*_SA1	*V.longisporum* *V.dahliae*	28.52
**Fusarium-specific amplification—** **EF1A (only with ASV-specific database)**								
Case11	Corn	*Fusarium graminearum, * *Fusarium avenaceum*	C2			*Fusarium tricinctum complex*		10.01
			C2			*Fusarium tricinctum complex*		23.89
Case12	Corn	*Fusarium sporotrichoides* *Fusarium graminearum* *Fusarium equiseti*	C2			*Fusarium fujikuroi complex*		28.56
			C2			*Fusarium incarnatum-equiseti complex*		67.20
Case13	Corn	*Kebatiellose* *Fusarium*	C2			*Fusarium incarnatum-equiseti complex*		1.11
			C4			*Fusarium*_SA89	*F.incarnatum equiseti complex* *F.sporotrichioides*	46.91
			C4			*Fusarium*_SA93	*F.asiaticum* *F.armeniacum* *F.boothii* *F.graminearum* *F.meridionale*	3.19
			C2			*Fusarium_serpentinum*		0.97
			C2			*Fusarium_sporotrichioides*		0.79
			C2			*Fusarium_sporotrichioides*		0.53
			C2			*Fusarium_tricinctum_complex*		7.37

## Data Availability

Supplementary Materials describe the Python notebooks used to generate ASV-specific databases and analyze environmental samples. All raw sequence files (FASTQ) have been deposited on NCBI. https://www.ncbi.nlm.nih.gov/sra/PRJNA1023220 (accessed on 1 August 2023).

## Supplementary Materials

The following supporting information can be downloaded at https://www.mdpi.com/article/10.3390/plants12213678/s1.

## References

[1] Mbareche H., Veillette M., Bilodeau G., Duchaine C. (2020). Comparison of the performance of ITS1 and ITS2 as barcodes in amplicon-based sequencing of bioaerosols. PeerJ.

[2] Bukin Y.S., Galachyants Y.P., Morozov I.V., Bukin S.V., Zakharenko A.S., Zemskaya T.I. (2019). The effect of 16S rRNA region choice on bacterial community metabarcoding results. Sci. Data.

[3] Abellan-Schneyder I., Matchado M.S., Reitmeier S., Sommer A., Sewald Z., Baumbach J., List M., Neuhaus K. (2021). Primer, Pipelines, Parameters: Issues in 16S rRNA Gene Sequencing. mSphere.

[4] Tedersoo L., Lindahl B. (2016). Fungal identification biases in microbiome projects: Fungal identification biases in microbiome projects. Environ. Microbiol. Rep..

[5] Schoch C.L., Seifert K.A., Huhndorf S., Robert V., Spouge J.L., Levesque C.A., Chen W., Bolchacova E., Fungal Barcoding Consortium, Fungal Barcoding Consortium Author List (2012). Nuclear ribosomal internal transcribed spacer (ITS) region as a universal DNA barcode marker for Fungi. Proc. Natl. Acad. Sci. USA.

[6] Bahram M., Anslan S., Hildebrand F., Bork P., Tedersoo L. (2018). Newly designed 16S rRNA metabarcoding primers amplify diverse and novel archaeal taxa from the environment. Environ. Microbiol. Rep..

[7] Comeau A.M., Vincent W.F., Bernier L., Lovejoy C. (2016). Novel chytrid lineages dominate fungal sequences in diverse marine and freshwater habitats. Sci. Rep..

[8] Callahan B.J., Mcmurdie P.J., Rosen M.J., Han A.W., Johnson A.J.A., Holmes S.P. (2016). DADA2: High-resolution sample inference from Illumina amplicon data. Nat. Methods.

[9] Prodan A., Tremaroli V., Brolin H., Zwinderman A.H., Nieuwdorp M., Levin E. (2020). Comparing bioinformatic pipelines for microbial 16S rRNA amplicon sequencing. PLoS ONE.

[10] Benson D.A., Cavanaugh M., Clark K., Karsch-Mizrachi I., Lipman D.J., Ostell J., Sayers E.W. (2013). GenBank. Nucleic Acids Res..

[11] Tateno Y. (2002). DNA Data Bank of Japan (DDBJ) for genome scale research in life science. Nucleic Acids Res..

[12] Quast C., Pruesse E., Yilmaz P., Gerken J., Schweer T., Yarza P., Peplies J., Glöckner F.O. (2013). The SILVA ribosomal RNA gene database project: Improved data processing and web-based tools. Nucleic Acids Res..

[13] DeSantis T.Z., Hugenholtz P., Larsen N., Rojas M., Brodie E.L., Keller K., Huber T., Dalevi D., Hu P., Andersen G.L. (2006). Greengenes, a Chimera-Checked 16S rRNA Gene Database and Workbench Compatible with ARB. Appl. Environ. Microbiol..

[14] Martin D., Rybicki E. (2000). RDP: Detection of recombination amongst aligned sequences. Bioinformatics.

[15] Deshpande V., Wang Q., Greenfield P., Charleston M., Porras-Alfaro A., Kuske C.R., Cole J.R., Midgley D.J., Tran-Dinh N. (2016). Fungal identification using a Bayesian classifier and the Warcup training set of internal transcribed spacer sequences. Mycologia.

[16] Nilsson R.H., Larsson K.-H., Taylor A.F.S., Bengtsson-Palme J., Jeppesen T.S., Schigel D., Kennedy P., Picard K., Glöckner F.O., Tedersoo L. (2019). The UNITE database for molecular identification of fungi: Handling dark taxa and parallel taxonomic classifications. Nucleic Acids Res..

[17] Pham V.H., Kim J. (2012). Cultivation of unculturable soil bacteria. Trends Biotechnol..

[18] Bokulich N.A., Kaehler B.D., Rideout J.R., Dillon M., Bolyen E., Knight R., Huttley G.A., Gregory Caporaso J. (2018). Optimizing taxonomic classification of marker-gene amplicon sequences with QIIME 2′s q2-feature-classifier plugin. Microbiome.

[19] Rognes T., Flouri T., Nichols B., Quince C., Mahé F. (2016). VSEARCH: A versatile open source tool for metagenomics. PeerJ.

[20] Zahariev M., Chen W., Visagie C.M., Lévesque C.A. (2018). Cluster oligonucleotide signatures for rapid identification by sequencing. BMC Bioinform..

[21] Pereira F., Azevedo F., Carvalho A., Ribeiro G.F., Budde M.W., Johansson B. (2015). Pydna: A simulation and documentation tool for DNA assembly strategies using python. BMC Bioinform..

[22] Parada A.E., Needham D.M., Fuhrman J.A. (2016). Every base matters: Assessing small subunit rRNA primers for marine microbiomes with mock communities, time series and global field samples: Primers for marine microbiome studies. Environ. Microbiol..

[23] Apprill A., McNally S., Parsons R., Weber L. (2015). Minor revision to V4 region SSU rRNA 806R gene primer greatly increases detection of SAR11 bacterioplankton. Aquat. Microb. Ecol..

[24] Bokulich N.A., Mills D.A. (2013). Improved Selection of Internal Transcribed Spacer-Specific Primers Enables Quantitative, Ultra-High-Throughput Profiling of Fungal Communities. Appl. Environ. Microbiol..

[25] Cobo-Díaz J.F., Baroncelli R., Le Floch G., Picot A. (2019). A novel metabarcoding approach to investigate Fusarium species composition in soil and plant samples. FEMS Microbiol. Ecol..

[26] Jeanne T., D’astous-Pagé J., Hogue R. (2022). Spatial, temporal and technical variability in the diversity of prokaryotes and fungi in agricultural soils. Front. Soil Sci..

[27] Bolyen E., Rideout J.R., Dillon M.R., Bokulich N.A., Abnet C.C., Al-Ghalith G.A., Alexander H., Alm E.J., Arumugam M., Asnicar F. (2019). Reproducible, interactive, scalable and extensible microbiome data science using QIIME 2. Nat. Biotechnol..

[28] Martin M. (2011). Cutadapt removes adapter sequences from high-throughput sequencing reads. EMBnet. J..

[29] Aurrecoechea C., Barreto A., Brestelli J., Brunk B.P., Cade S., Doherty R., Fischer S., Gajria B., Gao X., Gingle A. (2012). EuPathDB: The Eukaryotic Pathogen database. Nucleic Acids Res..

[30] Chen W., Radford D.R., Hambleton S. (2022). Towards Improved Detection and Identification of Rust Fungal Pathogens in Environmental Samples Using a Metabarcoding Approach. Phytopathology.

[31] Grinevich D., Harden L., Grinevich D.O., Callahan B.J. (2023). Serovar-level Identification of Bacterial Foodborne Pathogens from Full-length 16S rRNA Gene Sequencing. Microbiology.

[32] Boutigny A.-L., Gautier A., Basler R., Dauthieux F., Leite S., Valade R., Aguayo J., Ioos R., Laval V. (2019). Metabarcoding targeting the EF1 alpha region to assess Fusarium diversity on cereals. PLoS ONE.

