# Supplementary material for: ASVmaker: A New Tool to Improve Taxonomic Identifications for Amplicon Sequencing Data
Source: Plants (Basel). 2023 Oct 25;12(21):3678. doi: 10.3390/plants12213678 (PMC10647208; doi:10.3390/plants12213678)
Supplement: Supplementary file 1 [file plants-12-03678-s001.zip › ASVmaker_paper_archive/README.html]

README.md


# ASVmaker paper : data

This document describes how we create our database for the ASVmaker paper.
In order to give you a maximum of relevant informations, we removed the downloaded data, the
`.fasta` files and the `.json` temporary files generated by the tool.

By following this documentation, you should be able to regenarate the data.
We kept one Genus exemple per "origin" folder in order to understand the how
we renamed the files.

## 1. 16S

All the data generated during this step are available in the 16s/origin folder.

### 1.1 Retrieved FASTA files

**RNAcentral requests** :

- Erwinia : https://rnacentral.org/search?q=Erwinia
- Xanthomonas : https://rnacentral.org/search?q=Xanthomonas
- Pseudomonas : https://rnacentral.org/search?q=Pseudomonas
- Streptomyces : https://rnacentral.org/search?q=Streptomyces

**Silva requests** :

- LSU : https://www.arb-silva.de/fileadmin/silva\_databases/release\_138.1/Exports/SILVA\_138.1\_LSUParc\_tax\_silva.fasta.gz
- SSU : https://www.arb-silva.de/fileadmin/silva\_databases/release\_138.1/Exports/SILVA\_138.1\_SSUParc\_tax\_silva.fasta.gz

Then we use the script 16s/grepGENUS.sh on the downloaded data from SILVA to extract a FASTA file for each GENUS.

### 1.2. Processing

The primers used are in 16s/primers.

To create de database, the 16s/process.sh run for each GENUS the 16s/makeDB.sh for each origin database,
then the 16s/mergeDB.sh to merge both of the output database.

## 2. EF1a

All the data generated during this step are available in the ef1a/origin folder.

### 2.1. Retrieved FASTA files

**European Nucleotide Archive (ENA)**
Request :

- Fusarium : https://www.ebi.ac.uk/ena/browser/text-search?query=fusarium%20elongation%20factor%20alpha

### 2.2. Processing

The primers used are in ef1a/primers.

To create de database, the ef1a/process.sh run the ef1a/makeDB.sh on the origin database.

## 3. ITS

### 3.1 Retrieved FASTA files

All the data generated during this step are available in the ITS/origin folder.

**RNAcentral requests** :

- Colletotrichum : https://rnacentral.org/search?q=Colletotrichum
- Septoria : https://rnacentral.org/search?q=Septoria
- Ustilago : https://rnacentral.org/search?q=Ustilago
- Verticillium : https://rnacentral.org/search?q=Verticillium

**UNITE requests** :

- UNITE + INSD - 8.3 - Fungi : https://doi.org/10.15156/BIO/1281531

Then we used the script ./ITS/grepGENUS.sh on the downloaded data from UNITE to extract a FASTA file for each GENUS.

### 3.2. Processing

The primers used are in ITS/primers.

To create de database, the ITS/process.sh run for each GENUS the ITS/makeDB.sh for each origin database,
then the ITS/mergeDB.sh to merge both of the output database.
